# Right-Wing Psychedelia: Case Studies in Cultural Plasticity and Political Pluripotency

**DOI:** 10.3389/fpsyg.2021.733185

**Published:** 2021-12-10

**Authors:** Brian A. Pace, Neşe Devenot

**Affiliations:** ^1^Department of Plant Pathology, The Ohio State University, Columbus, OH, United States; ^2^Department of Comparative Literature, The Pennsylvania State University, State College, PA, United States; ^3^Department of Bioethics, School of Medicine, Case Western Reserve University, Cleveland, OH, United States

**Keywords:** psychedelics, authoritarianism, ecofascism, deep ecology, neoreaction, suggestibility, Jordan Peterson, capitalism

## Abstract

Recent media advocacy for the nascent psychedelic medicine industry has emphasized the potential for psychedelics to improve society, pointing to research studies that have linked psychedelics to increased environmental concern and liberal politics. However, research supporting the hypothesis that psychedelics induce a shift in political beliefs must address the many historical and contemporary cases of psychedelic users who remained authoritarian in their views after taking psychedelics or became radicalized after extensive experience with them. We propose that the common anecdotal accounts of psychedelics precipitating radical shifts in political or religious beliefs result from the contextual factors of set and setting, and have no particular directional basis on the axes of conservatism-liberalism or authoritarianism-egalitarianism. Instead, we argue that any experience which challenges a person's fundamental worldview—including a psychedelic experience—can precipitate shifts in any direction of political belief. We suggest that the historical record supports the concept of psychedelics as “politically pluripotent,” non-specific amplifiers of the political set and setting. Contrary to recent assertions, we show that conservative, hierarchy-based ideologies are able to assimilate psychedelic experiences of interconnection, as expressed by thought leaders like Jordan Peterson, corporadelic actors, and members of several neo-Nazi organizations.

## Introduction

The notion that psychedelic experiences can result in individual dispositional change has its roots in their association with the cultural and political upheavals of the late 1960's and 1970's. Because this specific time witnessed an explosion in countercultural fascination with widely available classical psychedelics like LSD, psilocybin, and mescaline, psychedelics have often been conflated with the progressive and radical politics of the moment: sexual revolution, the civil rights movement, and youth radical groups like Students for a Democratic Society or the Yippies. Yet psychedelic usage by modern and premodern culture predates this era, and different contexts did not produce individuals with a more progressive politics. In light of the nascent psychedelic renaissance and burgeoning psychedelic industry, the assumption that psychedelics lead to progressive politics deserves greater scrutiny—particularly given the hype around positive implications for society.

Prosociality and openness have both been shown under experimental conditions to be dramatically affected by psilocybin (MacLean et al., 2011; Griffiths et al., 2018). Among the core personality traits, openness—along with agreeableness—is linked to prosocial behavior (Kline et al., 2017).

Psychological research on political belief and ideology has sought to explain how individuals identify with political movements (e.g., conservatism) or political parties based on relative scoring in Big Five personality traits like openness (Caprara et al., 1999; Best, 2011; Gerber et al., 2011). Nour et al. (2017) investigated liberal-conservative leanings and the libertarian-authoritarian spectrum of drug users, using survey data (*n* = 893) from the recreational use of at least one classical psychedelic. Users of LSD, psilocybin mushrooms, mescaline, or ayahuasca were compared with cocaine and alcohol users as a non-psychedelic control population, finding that those with classical psychedelic experience were more likely to report less authoritarian beliefs than the non-psychedelic using controls. Shortly after, Lyons and Carhart-Harris (2018) addressed authoritarianism as an adjunct to an open-label psilocybin-assisted treatment-resistant depression therapy experiment, but with a much smaller sample size (*n* = 14), concluding that the seven participants who had psilocybin therapy reported lower authoritarian beliefs than before treatment or compared to the non-psilocybin control group. Results from these two studies constitute the only peer-reviewed evidence for the psychedelic experience predicting more liberal and less authoritarian values (see also the recent *n* = 1 observational correspondence by de Wit et al., 2021). Based on the dramatic clinical effects of psychedelics on openness and the putative reduction in authoritarianism, the media have constructed and proliferated an argument—at times, encouraged by researchers—that psychedelic therapy will lead to a more progressive, inclusive society (Gearin and Devenot, 2021). However, there are many historical counterexamples—those whose authoritarian views remained unaffected by psychedelic experience, or those who developed authoritarian views after or even with the assistance of a classic psychedelic—which the authors do not address. We suggest that the historical and cross-cultural records support the concept of psychedelics as “politically pluripotent,” non-specific amplifiers of the political set and setting (Lonergan, 2021).

Authoritarianism is a worldview with deep, cross-cultural roots which exalts obedience to hierarchical power relations above all else. On an individual level, people can display a range of authoritarian behaviors in their interpersonal relationships. Recent work by Zmigrod et al. (2020) has demonstrated that authoritarianism, along with nationalism, patriotism, and social conservatism, are predictable by low strategic information processing (e.g., planning, working memory capacity, and cognitive flexibility)—though each has a distinct cognitive trait signature. According to Arendt (1958):

The authoritarian relation between the one who commands and the one who obeys rests neither on common reason nor on the power of the one who commands; what they have in common is the hierarchy itself, whose rightness and legitimacy both recognize and where both have their predetermined stable place.

So long as hierarchy is held paramount, authoritarianism can take many forms. Typically, a natural law or hierarchy is posited and organized along multiple axes, such as racial identity, nationality, gender, and class (Crenshaw, 1991). Presently, white supremacy, patriarchy, heteronormativity, class, and imperialism constitute the dominant global infrastructure of authoritarianism (hooks, 2000). Elements of authoritarianism are organizing principles in military, penal, business, health, scholastic, and religious institutions (Foucault, 1995) and remain essential features of all political systems exercising state control.

## A Brief Review of Ideological Categorization and Nomenclature

In different historic contexts, ideologies have imposed meaning on both self and world, changing the relationship people have toward historically entrenched hierarchies, such as the divine right of kings during the feudal period. Some ideologies naturalize existing hierarchies, while others attempt to deconstruct them and envision alternative ways of distributing power. Many of today's current ideological conflicts can be traced to a basic division between supporters of some form of aristocracy and some form of shared democratic power. Democratic processes of debate, persuasion, individual autonomy, and egalitarianism are antithetical to authoritarianism.

The section following will briefly review a spectrum of the dominant political ideologies, moving from most general to more specific. A glossary of important terms and definitions can be found in Table 1. Historically, liberalism championed the rights of the merchant class and property-owning men, arguing for their exclusive enfranchisement. Liberalism emphasizes the economic liberty of individuals, which makes it the bedrock ideology of capitalism (Losurdo, 2011). Rooted in the philosophy and economic theory of John Locke and Adam Smith, liberalism rose during the Enlightenment as an alternative to hereditary monarchies (McLean and McMillan, 2009). As such, both conservatives and liberals believe in this sort of market-liberalism (O'Brien, 1993). The right wing-left wing divide takes its name from the different sides of French National Assembly of 1789, where supporters of the monarchy were seated on the right and those agitating for revolution on the left (Bobbio, 2016). The left, which opposes private control of the economy, includes socialist parties that have supported revolution and the seizure, socialization, and democratization of the means of production. The right, which supports private control of the economy, includes capitalist parties that center business interests and the private ownership and management of capital. In US politics, the terms left and right are often confused, since members of the Democratic Party are consistently labeled as “left-wing” even though they are technically center-right (Archer, 2007). The “libertarian” designation is also confusing: in US contexts, “libertarian” designates proponents of laissez-faire capitalism, yet this is a result of a historical co-optation of the term long used as a synonym for socialist, as in libertarian socialism (Diemer, 1997; Rothbard, 2007). US libertarianism can be delineated into right and left presentations, but while left libertarians are synonymous with libertarian socialists and some forms of anarchism, the far more common right libertarianism focuses on reducing government regulations and increasing the power of private industry (van der Vossen, 2019).

**Table 1:** Glossary.

**Term**	**Definition**
Alt-right	Shorthand for “alternative right,” the alt-right is an online rebrand of white nationalism, authoritarianism, patriarchy, and anti-Semitism associated with the politics message board of the website 4chan.
Authoritarianism	A hierarchical belief system that holds obedience to power to be the highest good.
Corporadelic	The term “corporadelic” was coined independently by Dr. Katherine MacLean (a former lead researcher and session guide for psilocybin research at Johns Hopkins University School of Medicine) and Brett Greene (co-founder emeritus of Psymposia) in 2019. Corporadelic refers to individuals and organizations who seek to monetize and privatize psychedelics using pharmaceutical industry strategies, such as generation of strong intellectual property (IP) and use of venture capital funds.
Dark Enlightenment	A twenty-first-century anti-egalitarian, anti-democratic, pro-corporate dictatorship, and authoritarian movement. Associated with NRx.
Deep Ecology	An environmental philosophy founded by Norwegian philosopher Arne Næss and American philosopher George Sessions in the 1970s and 1980s. Deep ecology argues that maladaptive environmental behaviors are symptomatic of Western culture's anthropocentric and utilitarian attitude toward nature, and that any meaningful behavior change depends on ameliorating this deep-seated sense of separation from nature.
Eco-fascism	An ideology that blames environmental degradation on overpopulation, immigration, globalization, and industrialization, to which it offers totalitarian “solutions” like ethnic cleansing. They believe that finite natural resources and environmental crises like climate change require the conservation and protection of those resources for the enjoyment and use of “white people.”
Ecofeminism	An intersectional philosophy and political movement that links ecological and feminist concerns, based on a critique of the patriarchal domination of society.
Ethnonationalism	An ideology that identifies one's nation with one's particular ethnic group while excluding other ethnicities. Ethnonationalists often scapegoat ethnic minorities for perceived problems facing the nation.
Fascism	The ideology of the historical Italian dictator Benito Mussolini and the two political parties he led as well as a modern, transnational ideology combining RWA and ultranationalism. It is characterized by the will to power and by domination (or extermination) of out-groups. Fascism is sometimes used interchangeably with modern forms of Nazism and white supremacy.
Intellectual Dark Web (IDW)	A loose affiliation between authors, academics, podcasters, and vloggers who oppose modern social justice activism, “cancel culture,” and political correctness.
Nazism	The ideology of both the historical National Socialist German Workers Party of the early twentieth century and of contemporary anti-Semitic, white supremacist, misogynistic admirers of Adolf Hitler.
Neoliberalism	Originally a theory of political-economic practices, neoliberalism has become a dominant and hegemonic global ideology that emphasizes individual responsibility and the erosion of the welfare state.
Neo-reaction (NRx)	A reactionary movement associated with the blog Unqualified Reservations. Since NRx expanded to become The Dark Enlightenment, the two terms are interchangeable.
QAnon	A far-right conspiracy movement most associated with the online forum 8chan (now 8kun) and with support for former President Donald Trump.
Race Science	The unscientific use of scientific narratives and vocabulary to justify white supremacist worldviews. It includes eugenics and attempts to reject the single-species designation of *Homo sapiens*.
Reactionary	A descriptor of persons or movements that seek a return to a real or imagined “golden era.” Reactionaries seek to regain lost power and are quick to act on perceived challenges to existing power structures from which they benefit.
Red Pill	A metaphor from the sci-fi movie *The Matrix* now commonly used to describe the process of radicalization to right-wing, white supremacist, and misogynistic ideologies. The first usage is attributed to the NRx blog Unqualified Reservations.
Social Dominance Orientation (SDO)	A personality type characterized by preferences for social conformity over individual autonomy and by submissiveness to authority figures.
White Supremacy	The belief that “white people” are a superior race that should dominate society for the purpose of society's optimal functioning, typically at the expense of racial and ethnic groups deemed “non-white” by adherents.

Although opposition to government regulation of enterprise has made frequent allies of right libertarians and conservatives, they differ in that conservatives support traditionalism while libertarians seek to reduce government intervention in the personal choices of individuals (e.g., opposition to the drug war), including corporate legal persons (van der Vossen, 2019). While both groups sympathize with free-market ideology, traditional conservatism adds a “concern for moral and communal factors” along with “a greater emphasis on nationalism and the authority of the state” (O'Sullivan, 1993). In *The Reactionary Mind: Conservatism from Edmund Burke to Donald Trump*, Robin (2018) offers a more psychological description, characterizing conservatism as “a meditation on—and theoretical rendition of—the felt experience of having power, seeing it threatened, and trying to win it back.” Adjacent to conservatives is the reactionary phenomenon: reactionaries seek a return to a historical or imagined social order (e.g., Make America Great Again). Whereas conventional conservatives believe in individual freedoms and meritocracy within traditional social relations, reactionary conservatives want the “correct” groups in power with the outgroups subordinate or excluded altogether (Nilsson and Jost, 2020).

Authoritarianism and libertarianism are expressed on both the left and right. Values cannot be neatly sorted into social and economic realms, nor do issues belong to any particular worldview. While there are often strong correlations between political leanings and stances on particular issues (e.g., care for the environment and progressivism), there are frequent enough departures (e.g., conservative love of country and outdoor recreation) that single-issue positions are poor proxies for defining ideologies.

The authoritarian right is populated by a constellation of movements that includes “radical-right, anti-liberal democracy actors to extreme-right, anti-democracy ones” (Forchtner, 2019), as well as ultranationalist, fascist, and neo-Nazi groups (Davies and Lynch, 2002). Right-wing authoritarianism (RWA) reflects “preferences for social conformity over individual autonomy,” whereas social dominance orientation (SDO) emphasizes “preferences for social hierarchies as opposed to egalitarianism” (Cohrs, 2012). Both RWA and SDO are associated with “system justification,” defined as “the tendency to defend and bolster the societal status quo” (Nilsson and Jost, 2020). Conservatism is continuous with ultranationalism, which goes beyond the shared history, language, and traditions associated with conventional nationalism by elevating one's group identity above all other nations through xenophobic animus (Rydgren, 2018). When a particular ethnicity within a nation is exalted above others within and without the nation, ethnonationalism results (Bonikowski, 2017). Within ethnonationalist worldviews, ethnic minorities are often scapegoated for problems facing the nation—both concrete and fictitious (Forchtner, 2020).

The phenomenon of scapegoating is of particular importance when considering fascism and Nazism (a subtype of fascism). Fixation about internal enemies is cast in terms of sickness and disease in an organic politic where the nation, its peoples, and borders are conceptualized as a living body, such as in the 1940 Nazi film *Der ewige Jude* (*The Eternal Jew)*, where Jews are compared to parasites and rats. Weakness is openly disdained by Nazis and fascists, as reflected in the embrace of social Darwinisim and eugenics in both past and present day (Black, 2012). Empathy is affected when problems and people are framed as pathogens, where empathy for an outgroup is seen as analogous to skipping radiation treatment out of empathy for cancer cells. The possibility of increasing empathy for one's in-group problematizes universal claims about psychedelics increasing empathy for all people in the research literature (Blatchforde et al., 2021). In both fascist and Nazi ideologies, out-groups are cast as weakening the nation and robbing the master race of its destiny as the true heir to the Roman Empire. The fascist, anti-materialist mythologizing of past, present, and future rejects intellectualism and intellectuals (Sternhell, 1987)—indeed anything that stands in the way of victory. Fascism can take many forms and vary in degree—hence the term “crypto-fascist,” which refers to those concealing fascist sympathies or “hiding their power level” (ContraPoints, 2017)—but the guiding principle has always been the will to power and domination (or extermination) of out-groups. While fascists will form alliances when convenient, they will easily turn on perceived out-groups that formerly served their interests when conditions allow for it.

## Power Tripping

Anyone positing that psychedelics—therapeutically or recreationally applied—result in more liberal or anti-authoritarian views must contend with many contemporary and historical counterexamples (Jacobs, 2020; Johnson and Yaden, 2020; Pace, 2020). There are those who, after one or many psychedelic experiences, held on to pre-existing authoritarian views; and those who developed an affinity for authoritarianism after—or even as a result of—a psychedelic experience. Anthropologist Nicholas Langlitz described his own experience with what he called Rightist Psychedelia:

Today, the classmate who gave me my first LSD trip for my eighteenth birthday does what he can to limit social inclusion of immigrants in Germany. He came to represent the far-right populist party *Alternative für Deutschland* in the parliament of North Rhine-Westphalia. Consciousness expansion and rightist thought have never been mutually exclusive and they are currently reconnecting. (Langlitz, 2020)

In both the experimental and naturalistic studies that claim psychedelics make people more liberal, it is far more likely—and parsimonious—to surmise that environmental factors account for the change toward more progressive views. For example, the inclusion of ayahuasca in Nour et al. (2017) alone skews the survey population to those already so open to novelty as to seek out psychedelic experience in what is most commonly an Indigenous, jungle context. Similar points adhere with the inclusion of mescaline. People predisposed to xenophobia and white supremacy are likely to self-select out of these user populations. However, globalized, source-blind supply chains divorce certain chemicals from their indigenous contexts—darknet and internet-enabled gray market purchases may render psychedelic analog research chemicals or extracted DMT more palatable to authoritarian-leaning users than the DMT and MAOI-containing ayahuasca brew with all of its attendant cultural “baggage” and destination jungle settings.

Prior to the recent wave of studies on psychedelics and liberalism and nature-relatedness (Forstmann and Sagioglou, 2017; Nour et al., 2017; Lyons and Carhart-Harris, 2018; Kettner et al., 2019), Piper (2015) addressed several historical and contemporary examples that demonstrate that intimate knowledge of classical psychedelics have coexisted with far right, authoritarian views since the very beginning of psychedelic exploration by modern Western investigators. For instance, fascist philosopher, occultist, and author of the introduction of the Italian version of *The Protocols of the Elders of Zion*, Julius Evola, was known to experiment with and write about drugs, and he is an influential figure for the alt-right today. Indeed, the first botanical collection of *Teonanácatl* was facilitated by the Austrian botanist and “ardent Nazi” Blas Paul Reko, who corrected Richard Evans Shultes' earlier designation of the Aztec sacrament as peyote (Schultes, 1939; Davis, 1996):

Aristocrats, bankers, German right-wing ex-military men, not to mention the CIA and the Nazis: From the 1930s to the 1960s, it is a little acknowledged fact that one of the principal sources of interest in psychedelics, aside from the interest of researchers working on specific therapeutic uses, was people of conservative or right-wing orientation. (Boon, 2002)

The early days of non-therapeutic LSD use was a consciously exclusive and elite—rather than democratic—activity, typified by the private sessions hosted by Dr. Hofmann with Ernst Jünger described in detail in the chapter entitled, “The Radiance of Jünger” in his autobiography, *LSD: My Problem Child*. Most known for his literary works, Jünger was a Wehrmacht officer and censor for the Nazis in occupied France during WWII; he also coined the term “psychonaut” to describe the work of chemist and mescaline explorer Arthur Heffter (Jünger, 1970; Piper, 2015).

Continuing this lineage into the present, neo-Nazis with histories of psychedelic use do not evidence decreased authoritarianism as a result of such experiences. In keeping with the mystical elements of both Nazism and fascism, contemporary fascist beliefs often overlap with or incorporate elements of occult or Satanic practice (Lavin, 2020; Owen, 2020). The Base, an international neo-Nazi group, integrated collective LSD use into a neo-Pagan, male-bonding ritual involving the beheading of a stolen ram (Makuch and Lamoureaux, 2020b). Members of The Base were later arrested on weapons charges and discovered to be attempting to extract DMT (Makuch and Lamoureaux, 2020a). The Base had been under investigation for planning the murder of an antifascist couple and their child. Andrew Thomasberg attended the August 2017 “Unite the Right” rally in Charlottesville, Virginia with the neo-Nazi group Vanguard America. He later became a recruiter for Atomwaffen Division, an occult neo-Nazi organization linked to at least five murders (Weiner, 2020). Thomasberg was arrested for weapons charges and, while in custody, praised the murder of nine black churchgoers by Dylann Roof. In released text messages, Thomasberg stated, “There's nothing more Aryan [sic] than ethneogenic [sic] drug use,” and described a deep familiarity with LSD, mushrooms, and several other scheduled drugs (Owen, 2019). “Unite the Right” was organized by Jason Kessler, a member of the Proud Boys (Barnes, 2017); although their then-leader, Gavin McInnis, was not in attendance, he applied his extensive drug history (Gollner, 2021) to publicly advise his followers on how to navigate challenging psychedelic experiences in a 2016 episode of his talk show (Flutez, 2016). The Atlantic reported that Andrew Anglin, the Columbus, Ohio native founder of the neo-Nazi website, *The Daily Stormer*, had extensive experience with classical psychedelics such as LSD and psilocybin as a teenager before his metamorphosis into a fascist propagandist (O'Brien, 2017).

Stormfront is the oldest hate site on the internet, focusing on white nationalism, Holocaust denial, and neo-Nazi organizing. A simple search for “psychedelics” on the Stormfront forum turns up pages of comments—both positive and negative—discussing the science, therapeutic value, and cultural impact of these drugs:

**Originally posted by Puddintane:**

I wonder what LSD does to racial awareness. People who use it talk about feeling more connected, but how does that translate to racial awareness? Does anyone have any observations about this?

**Forum Member:** w41n4m01n3n

**Join Date:** Apr 2017

**Location:** Embassy of the 4th Reich in Finland

**Posts:** 8,581

Well I can tell you this much: I have tried LSD several times and it certainly did not make me a liberal idiot. You can go through my posts and see for yourself. There are many studies verifying what most people had already suspected: LSD is not dangerous to one's physical health. Whether it is damaging to one's psychological well-being, depends on the person, dose and circumstances. Long-term negative psychological effects are extremely rare. LSD was invented in 1938 by Swiss chemist Albert Hofmann (whom I have met in real life in 2006 on his 100th birthday in Basel, Switzerland), but its psychoactivity wasn't known until 1943.... But I also think, that we shouldn't be discussing these matters this much on Stormfront. I don't think it is the right place for such matters. It could give the wrong message (to youngsters in particular). (Stormfront, 2017)

Psychedelics have a place in the cutting edge of the radical right, both digitally and as literal boots on the ground. Frederick Brennan was inspired to create the free speech absolutist website 8chan while on a psilocybin mushroom trip (McLaughlin, 2019). Long a haven for digital neo-Nazis and other antisocial posters, 8chan has since been disavowed and shut down—chiefly through the efforts of Brennan—after the entrance of mass shooters on the board. Before the shuttering of 8chan and its re-emergence as 8kun, three mass shooters posted their manifestos on 8chan prior to carrying out their murders (Harwell, 2019). The documentary series *Q: Into the Storm* followed Brennan's crusade against his former business partners, Jim and Ronald Watkins, and makes the case that the father-and-son duo are behind the Q phenomenon (Hoback, 2021). “Q-drops” had their own psychedelic tenor, with the hashtag #followthewhiterabbit being associated with elaborate conspiracy theories about elite child molestation rings and the psychoactive (but, despite popular culture references, not psychedelic) substance adrenochrome. Incitements from President Trump, Alex Jones, and the drip of Q-drops led to the storming of the US capitol building. Prominently included in their ranks was the self-described Q-Shaman (AKA Jake Angeli Chansley, Yellowstone Wolf) and William Watson, both individuals with deep and extensive ties to the psychedelic community (Evans, 2021; Pace, 2021a,b). Beyond multiple and clear intersections of the psychedelia with the cutting edge of right-wing activism, right-wing conspiracy seems more than able to spring from native psychedelic soil. The name and reputation of Timothy Leary's Castalia Foundation was recently appropriated to praise President Trump and promote Q-reminiscent child exploitation conspiracies, complete with a classic anti-Semitic depiction of Rick Doblin (Castalia Foundation, 2020; Hausfeld, 2021).

Along with hyper-association and boundary dissolution, a core element of the subjective experience of psychedelics is meaning-enhancement (Hartogsohn, 2018). Combined with the influences of set and setting, a psychedelic experience can lead to shifts in worldviews—and even religious and political ideologies—but not in a consistent, directional manner. A more complete model draws from the literature on radicalization where destabilizing experiences—such as the loss of a loved one or subjection to violence or abuse—can induce what is known as a “cognitive opening,” which calls into question an individual's extant worldview, marking the first step on a potential path to extremism (McCauley and Moskalenko, 2017). In such cases, community support for radical belief (i.e., the setting and other extrapharmacological factors) is an important contributing factor. The antifascist investigative journalism outlet *Bellingcat* surveyed online accounts of 75 neo-Nazis on their process of radicalization and found that four cited an LSD experience as foundational to their “red-pilling” to Nazi beliefs (Bellingcat, 2018).

User Europa is a typical example of this trend. He claims his interest in Nazism started in childhood, with his dad watching Hitler documentaries “every day.” Europa carried this interest into adulthood, watching Hitler documentaries and speeches while taking LSD. This convinced him to start “researching” Nazism which, eventually, inspired him to become an activist.

This sensitivity to the “initial rhetorical conditions” (Doyle, 2011) of set and setting is consistent with the measurable impact of suggestibility on psychedelic experiences. In a study of the putative suggestibility-enhancing effects of LSD, for instance, Carhart-Harris and colleagues found that LSD increased the subjective intensity of researcher-suggested sensations that included one's arm becoming heavier, reexperiencing oneself as a child, and drinking especially refreshing water (Carhart-Harris et al., 2015). McGovern et al. (2021, Preprint) explore the implications of this suggestibility for the ways that beliefs interface with psychedelic experience, noting that “[I]f psychedelics change ‘beliefs’, they do so largely through experimenter suggestion and the pre-existing psychology of the individual.” Dupuis argues that even hallucinatory images are influenced by extrapharmacological factors including “cultural priors and acquired knowledge” (Dupuis, 2021). In the study of Indigenous cultures, anthropologists and other researchers have long asserted that the suggestibility-enhancing attributes of psychedelics are key ingredients in fostering social cohesion and reinforcing group norms.^1^ Dobkin de Rios and Rumrrill (2008) have linked this phenomenon to the psychological concept of “bounded rationality,” defined as the human tendency to act based on social influence. They further remarked that when psychedelics are used outside of a normative context, users may develop antinomian ideas (Grob and Dobkin de Rios, 1992; Dobkin de Rios and Rumrrill, 2008). Given this link between psychedelics and suggestibility, which had already been noted by the early wave of researchers in the twentieth century, contemporary commentators should be skeptical of claims that psychedelics lead to any specific, essentialized outcomes—including the recent claims about psychedelics increasing environmental concern and liberal politics.

Psychological research has also demonstrated that purported attributes of psychedelic experiences—such as increased feelings of gratitude—are themselves sensitive to the context of set and setting variables. Although gratitude has been referenced to explain the prosocial benefits of psychedelics (Gandy, 2019; Moreton et al., 2020), a recent paper by Tong and colleagues argues that gratitude can facilitate authoritarian obedience under specific context conditions. In their study, participants who were induced to feel gratitude were more likely to obey commands to grind worms in a grinder compared to those without gratitude priming. The authors note that “gratitude could increase susceptibility to social influence, making the grateful person more likely to align with social conventions and directions…. [T]he grateful person is prone to keep in line with social forces, the consequences of which could sometimes be undesirable” (Tong et al., 2020). Similar mediating variables have been highlighted in the literature on mindfulness, which—like gratitude—is conventionally associated with prosocial behaviors and positive emotional adjustment. In a 2021 paper, however, mindfulness induction was shown to decrease prosocial behavior among participants who were primed with self-construals that emphasized independence rather than interdependence, which suggests that prosocial outcomes are the result of specific (set and setting) conditions rather than an inherent quality of mindfulness-based attention (Poulin et al., 2021, Preprint).

## Deep Ecology and ECO-Fascism: the Problem of Hierarchy

Recent correlational studies have associated psychedelic use with pro-environmental beliefs and behaviors. On the basis of preliminary evidence linking experiences of “unity and connection with one's environment to empathy-related phenomena and pro-environmental behavior,” Forstmann and Sagioglou (2017) sought to establish whether lifetime psychedelic experience correlated to increased nature relatedness and pro-environmental behavior. The authors focused on 12 “headline” pro-environmental behaviors within four key areas of “everyday” concern: domestic resource consumption, waste behavior, transportation use, and eco-friendly shopping. Based on results from 1487 online survey participants, they found evidence that psychedelic experience correlated with an increased and lasting sense of interconnection with nature. Furthermore, this “ecocentric” identification—wherein nature is conceived of as an “integral” part of one's self-concept—also correlated with higher reports of pro-environmental behaviors. This outcome supports the findings of Nour et al. (2017), who found that lifetime psychedelic use correlated with nature relatedness in addition to liberal political views.

These correlational studies draw on the preexisting psychological literature on “nature relatedness” and the associated Nature Relatedness scale, a psychological tool used to measure “the affective, cognitive, and experiential aspects of individuals' connection to nature” (Nisbet et al., 2009). To date, there appears to be little overlap between this field and the study of eco-fascism and other right-wing movements wherein nature relatedness plays a prominent ideological role, including “eco-naturalism (the natural world as a blueprint for the social order), eco-organicism (the Volk/people as an ecosystem) and eco-authoritarianism (the need for a strong state to deal with the environmental crises of our time)” (Forchtner, 2019).

In describing their predictions for their study, Forstmann and Sagioglou expected measures of political conservatism to negatively predict both psychedelic usage and pro-environmental behavior, on the basis of two citations in the published literature. In the first paper by Kurzban et al. (2010), conservatives were associated with negative views on “recreational drug use.” In the second paper by Schultz and Stone (1994), right-wing authoritarianism was “highly negatively correlated” with environmental concern, based on a study of reactions to the development of a new power plant and a separate questionnaire about public environmental views more generally. Commenting on the paper by Schultz and Stone, however, J. Christopher Cohrs notes that anti-environmentalism is “not inherent to RWA or SDO” (Cohrs, 2012). In discussing their findings, Schultz and Stone even predict that this negative correlation between authoritarianism and environmental concern is likely to disappear over time. Writing in 1994, they noted that the “established authorities” within local social hierarchies—including public political figures and capitalist developers—overwhelmingly favored economic growth over environmental protectionism, and that authoritarian-minded individuals likely took their cues from these positions. Schultz and Stone argue, however, that this preponderance of progrowth ideology among the ruling elites is unlikely to last, given that climate change and its associated environmental catastrophes will become increasingly difficult to ignore: “If this analysis is correct, as protection of the environment receives more and more attention and support, the new environmental paradigm will become the [new] conventional wisdom…. Thus, we would expect a decline over time in the negative relationship between authoritarianism and environmentalism” (Schultz and Stone, 1994).

Nearly three decades after Shultz and Stone published their initial findings, there are indications that this shift in environmental concern among right-wing authorities is already well underway. The effects of climate change have become increasingly obvious and disruptive since 1994, with observable increases in extreme weather and climate events including wildfires, flooding, droughts, storms, and heatwaves (Ummenhofer and Meeh, 2017). Climate change is projected to increase the intensity, frequency, and duration of extreme weather events in the future, with corresponding increases in vector-borne diseases, food and water insecurity, and property damage as the century progresses (Pastor-Paz et al., 2020; Rocklöv and Dubrow, 2020; Koren et al., 2021). In light of these trends, environmentalism is expected to become “one of the central political issues of the next decades” across the political spectrum, including amongst the far right (Rueda, 2020).

Historical evidence likewise suggests that the current common association of environmentalism with the values of “progressivism, liberalism, equalitarianism, and respect for democracy” is a matter of historical contingency rather than necessity (Rueda, 2020). As Michael E. Zimmerman points out, many postwar leftists in the mid-twentieth century were “suspicious of environmentalism: they feared that it was somehow linked with racist, reactionary politics, which justified oppressive power arrangements on the basis of their ‘natural’ character” (Zimmermann, 1993).

As Forstmann and Sagioglou (2017) emphasize, social psychological research indicates that the “self-other overlap” characteristic of nature relatedness involves both “an incorporation of the target's features into the self” and an “ascription of one's own characteristics to the target.” This two-way process of incorporation and projection is thought to increase the “empathic concern” felt for the target or “other” (in this case, nature), since the “other” is experienced as an extension of the self. This notion represents the theoretical basis for deep ecology, which holds that subjective identification with nature increases the intrinsic motivation to care for the environment.

Founded by Norwegian philosopher Arne Næss and American philosopher George Sessions in the 1970s and 1980s (Devenot, 2016), deep ecology argues that the maladaptive environmental behaviors that fuel climate change are symptomatic of “Western culture's anthropocentric, dualistic, and utilitarian attitude toward nature” (Zimmermann, 1993). Deep ecologists argue that without addressing this deep-seated sense of separation from nature, environmental “reform” movements that seek to moralize pro-environmental behavior are unlikely to furnish the commitment to meaningful behavior change that will be necessary to avert climate catastrophe. Although the philosophy of deep ecology developed prior to the psychological literature on nature relatedness, psychedelic researchers have cited deep ecology to articulate a theoretical mechanism for the correlation between psychedelic experiences and pro-environmental concern and behaviors (Lawlor, 2008; Luke, 2013). The synergy is especially evident in deep ecology's emphasis on the importance of non-ordinary states of consciousness for cultivating an ecocentric self-concept, beyond the ordinary sense of separation from nature:

While emphasizing scientific claims about the interrelatedness of all things, deep ecologists insist that intellectual conclusions alone are not sufficient to bring about a basic shift in one's attitude toward nature. Such a shift requires a change of consciousness, an intuitive sense of identification with all things. Arne Naess [sic] argues that such intuition leads us to reject the idea that we are “in” the environment, as if we were surrounded by something basically different from us. An intuitive sense of wider identification enables us to cultivate [“]*the relational, total-field image*.[”] (Zimmermann, 1993)

Given the relevance of deep ecology for the psychedelic research literature on nature relatedness, it is worth considering how deep ecological constructs can be recuperated for authoritarian purposes based on a hierarchical social order. Historical examples of this recuperation necessarily problematize the commonplace association of nature relatedness with empathy and liberal political values within the psychedelic research literature. As Michael E. Zimmerman has noted, intellectual predecessors of the deep ecology movement include the German philosopher Martin Heidegger along with other supporters of National Socialism and the Nazi party. Like the deep ecologists that came after him, Heidegger diagnosed the need for humanity to transition away “from an anthropocentric, dualistic, and utilitarian understanding of nature” through an “ontological shift” in order to avert ecological crisis (Zimmermann, 1993). Zimmerman notes the compatibility of this perspective with elements of National Socialism, which Robert Pois has characterized as a “religion of nature”:

According to Pois, [“]the National Socialist call[s] for the establishment of a utopian community, the *Volksgemeinschaft*, rooted in a perceived natural order…. [This ideology emphasizes the] overcoming of alienation, not through some hideous form of class war, but rather through a revolution of consciousness, the result of which would be a new sense of rootedness and belonging.[”]

Jules Evans has likewise pointed to the Nazi professor of botany, Ernst Lehmann, who stated:

We recognize that separating humanity from nature, from the whole of life, leads to humankind's own destruction and to the death of nations. Only through a reintegration of humanity into the whole of nature can our people be made stronger... This striving toward connectedness with the totality of life, with nature itself, a nature into which we are born, this is the deepest meaning and the true essence of National Socialist thought. (Evans, 2020)

The possibility for feelings of interconnection with nature to slip into justifications of hierarchy and social domination has long been a feature of ecofeminist critiques of deep ecology. Although deep ecology and ecofeminism are compatible in principle, ecofeminists have expressed concern that certain interpretations of deep ecology's central tenets risk legitimizing authoritarian forms of social power. Regarding their similarity, ecofeminism shares deep ecology's contention that ecological destruction is symptomatic of a central misapprehension about the nature of the self and its relationship to the environment. Both philosophies attribute this misapprehension to the effects of living under industrial capitalism, wherein “individuals are seen as discrete, atomistic beings, who must be compelled, through the use of reason, to behave in moral ways” (Kheel, 1991). Skeptical of any attempt to improve environmental behavior through abstract, ethical moralizing, both deep ecologists and ecofeminists emphasize the importance of direct experiences of interconnection in order to shift innate levels of environmental concern. As Warwick Fox notes, “Deep ecologists agree...that ‘human beings are more deeply moved by the way they experience their world than by the claims ethics makes on them”’ (cited in Kheel, 1991).

In the essay “Ecofeminism and Deep Ecology: Reflections on Identity and Difference,” Marti Kheel explores three case studies involving eco-philosophers who describe an expanded sense of self-identification that encompasses the natural world, as expressed specifically through the act of hunting animals. In each of these instances, Kheel argues, the sense of interconnection allows the hunter to legitimize the hierarchical relationship between self and animal as “natural” and healthy, such that the self's preferences and priorities are projected onto the “other” regardless of the animal's own interests or preferences:

Deep ecologists argue that a widened identification will ensure that one will want to minimize harm to individual beings in that they will be viewed as part of the larger ecological Self. However, it is clear from the above examples of self-realization, that for these men this was not the case…. The mind-set that they reveal is that of a psychological instrumentalism in which the animal is seen, not as a unique, living being, but rather, as a means of achieving a desired [heightened] psychological (or psycho-biological) state…. The significance of the reduction of the animal to object status is that the relationship to the animal becomes more important than the animal itself. The feelings of yearning for union, the urge to “outwit”—all these take precedence over the living being that is killed. The animal is swallowed up in the act of merging. Even the death of the animal is considered incidental—a byproduct of the more important desire that finds its expression in the hunt…. When deep ecologists write of expanding the self, ecofeminists must be prepared to examine more deeply the unconscious drives that fuel the self that one seeks to expand…. As we have seen, the danger of an abstract identification with a larger “whole” is that it fails to recognize or respect the existence of independent, living beings…. Ecofeminist philosophy must be wary of a holist philosophy that transcends the realm of individual beings…. Individual beings must not be used in a kind of psychological instrumentalism to help establish a feeling of connection that in reality does not exist. (Kheel, 1991)

Although Kheel's analysis specifically focuses on descriptions of hunting animals by ecophilosophers adjacent to the deep ecology movement, this central dynamic is transferable to any interpersonal scenario where the other is subsumed within a “natural” or “organic” social hierarchy.

As Daniel Rueda documents in his 2020 analysis of the growing threat of neo-ecofascism, this phenomenon is already becoming apparent amongst prominent figures in the alt-right. The alt-right has been defined as “a constellation of right-wing forces...animated by political commitments to white nationalism or ultranationalism, authoritarianism and rejection of democracy, gender traditionalism, hatred of the left and liberalism, and antisemitism” (Taylor, 2020). Although the movement is heterogeneous, it is “united by the belief that...[s]traight white western men are situated at the apex of...civilizational hierarchy,” and that any counter-movements seeking to remedy inequality and oppression—such as feminism or anti-racism—threaten the very foundations of civilization by opposing that natural order (Taylor, 2020). Notably, the alt-right illustrates how this sense of a differential social hierarchy can still be compatible with a nature connectedness similar to that espoused by deep ecology. Richard Spencer, an avowed neo-Nazi who coined the term “alt-right” in 2008, included an evocation of nature connectedness in his manifesto for the “Unite the Right” rally in Charlottesvile, VA: “We are a special part of the natural order, being in it and above it. We have the potential to become nature's steward or its destroyer. European countries should invest in national parks, wilderness preserves, and wildlife refuges, as well as productive and sustainable farms and ranches. The natural world—and our experience of it—is an end in itself” (Spencer, 2017; Rueda, 2020; Vanderheiden, 2020). In this and similar instances, nature is portrayed as a birthright and constituent element of the social elites, while those lower down the social hierarchy are portrayed as “deviants” who threaten the integrity of the moral and natural order.

The historical evidence of ideological compatibility between interconnection and hierarchy casts doubt on researchers' claims that psychedelics are inherently destabilizing to existing hierarchies. Roland Griffiths—founding Director of the Johns Hopkins Center on Psychedelic and Consciousness Research—expressed this common perspective in an interview with Michael Pollan: “There is so much authority that comes out of the primary mystical experience that it can be threatening to existing hierarchical structures” (Pollan, 2018). Others have suggested that experiences of interconnection inherently lead to universal increases in care and concern, without hierarchical gradations: “Psychedelic moral enhancement's goal…is to create individuals better able to engage in interpersonal interactions for altruistic purposes because they have glimpsed the unique value of each part of the interconnected whole of the universe” (Stankey, 2020). Some researchers have even theorized a connection between psychedelic changes to political beliefs and the neurological impact of psychedelics on cognitive hierarchies, as reported in *Vice*: “‘The general take-home message is that the psychedelic state is seemingly the breakdown of hierarchical organization in the brain,’ [Alexander] Lebedev says. In a sense, this breakdown could somehow be mirrored in people's shift in political beliefs, he suggests. ‘In that way, it's not completely surprising that we see these changes after such a session”’ (Henriques, 2018). In the next section, we will explore the specific example of Jordan Peterson to show how the concepts of interconnection and hierarchy can be intertwined.

## Lobsters All the Way Down: Jordan Peterson on Psychedelics

Jordan Peterson bills himself as an individualist, classical liberal, but Peterson has been characterized as an “extreme conservative” thought leader for the “New Right,” and his work—especially his attacks on “intersectional politics and liberal egalitarianism”—carries strong appeal among “Evangelical Christians, paleoconservatives and white supremacists” in particular (Nicholls, 2019). Evidence from both critical (Lewis, 2018) and sympathetic (Finkelstein, 2019) sources document Peterson and the associated Intellectual Dark Web (IDW) community as gateways to the alt-right. The Jungian psychologist became a conservative icon by railing against an expansion of Canadian human rights legislation Bill C-16, which he characterized as the first step toward compelled speech and totalitarianism. Peterson “found a way to monetize SJWs [Social Justice Warriors]” (Rogan, 2017) by arguing (incorrectly) that the bill would result in the arrest and imprisonment of faculty who refused to use transgendered students' correct pronouns. Peterson's dire predictions about the bill never coming to pass did not result in his followers deserting him, which is consistent with the work of Garrett and Bond (2021). In a longitudinal study of viral news stories, the authors demonstrated that US conservatives are more susceptible to political misconceptions, particularly when they benefit the in-group. Peterson abandoned academia to start a career as a conservative thought leader and has spoken on a wide range of topics, from the archetypes of The Lion King to arguing in support of traditional gender roles and sexual conservatism.

However, Peterson's interactions with the far right have made it clear that he believes in and defends a race-based intelligence hierarchy. While a guest on white supremacist, right-libertarian Stefan Molyneux's podcast, Peterson specifically appealed to Herrnstein and Murray's *The Bell Curve* (Herrnstein and Murray, 1994) to support his views:

The IQ literature reveals that which no one would want to be the case, which is that there are profound and virtually irremediable differences in people's cognitive performance and that those differences have a very solid biological and heritable basis. No one wants to hear that. They don't want to hear that it's biological. They don't want to hear that it's heritable. They don't want to hear it's permanent…. [T]he gender differences in IQ look relatively trivial—but there are differences in ethnicity that don't look trivial. The Ashkenazi Jews, for example, have on average a 15-point advantage over the rest of the Caucasian population, which is sufficient to account for the radical over-representation in positions of authority and influence and productivity…. We have a really profound problem, then again, that no one's willing to talk about and that's the cognitive stratification of society. And of course, that was laid out to some degree in *The Bell Curve* back in the 1990's. (Perez, 2020)

*The Bell Curve* is a work of scientific racism that has been thoroughly rebutted by scholars from multiple disciplines (Konnor, 2003; Marks, 2010), notably including evolutionary geneticist Stephen Jay Gould, who added two chapters to the second edition of his book, *The Mismeasure of Man* (Gould, 1996) to debunking Herrnstein and Murray's race science claims. Repeated efforts to push “race realist” narratives have been consistently dismissed as unscientific projects of a white supremacist ideology—and contrary to analysis of both the human genome and human migration patterns. A recent letter to *Philosophical Psychology* by Larsen et al. (2020) is emblematic:

An abundance of evidence about the human evolutionary lineage shows that no human groups were ever phylogenetically distinct in the way that racial realism posits. In other words, the human species never persisted in multiple sufficiently isolated “pure” groups for such a prolonged time that it would, evolutionarily speaking, make sense to talk about our species being divided into biologically distinct populations. Indeed, migration in and between continents and regions was never a one-way movement, but, rather, an intricate multi-way network of substantial intersections and interactions. Because of this mobility, the genetic variation within the human species is much more complex than presumed or predicted by racial realism.

For Peterson, as for Richard Spencer, the existing social hierarchy of Western civilization must be protected at all costs from the egalitarian “social justice warriors” who seek to upend it. Peterson's writings naturalize and mythologize (Brooks, 2020) this hierarchy as the bedrock of civilization, which functions to protect humanity from the outlying forces of chaos, madness, and meaninglessness:

The order within the chaos and order of Being is all the more “natural” the longer it has lasted. This is because “nature” is “what selects,” and the longer a feature has existed the more time it has had to be selected—and to shape life. It does not matter whether that feature is physical and biological, or social and cultural. All that matters, from a Darwinian perspective, is permanence—and the dominance hierarchy, however social or cultural it might appear, has been around for some half a billion years. It's permanent. It's real. The dominance hierarchy is…a near-eternal aspect of the environment…. We were struggling for position before we had skin, or hands, or lungs, or bones…. Dominance hierarchies are older than trees. (Peterson, 2018)

Peterson's thinking provides a philosophical legitimation for social dominance orientation (SDO), defined in terms of an individual's enduring preference for group-based hierarchies and inequalities. For Peterson, all of humanity is “interconnected” *via* the “natural” sorting process that shuttles individuals to the appropriate status within this hierarchy, which he grounds biologically in the serotonergic system that we hold in common with lobsters and other lifeforms: “The part of our brain that keeps track of our position in the dominance hierarchy is therefore exceptionally ancient and fundamental. It is a master control system, modulating our perceptions, values, emotions, thoughts and actions.” As he lays out in *12 Rules for Life*, Peterson describes the “dominance hierarchy” as among the “most real” and “ever-present” aspects of society (Peterson, 2018).

Peterson's interest in the serotonergic basis of hierarchy has recently extended to a fascination with the effects of psychedelics on the serotonergic system, as Peterson laid out in an extended podcast discussion with Johns Hopkins' Roland Griffiths:

I think your work is of...incalculable import.... I actually can't believe how important this is…. I've been studying the psychedelic literature for 20 years…in as deep a manner as I can possibly manage, and every time I think I have some grasp on how important it is, I learn something else and I think, “Oh ho! It's *way* more important than I thought it was!” It's—it's of *crucial* significance. It's, it's of central—it's literally of central significance. [Repeating:] It's *literally* of central significance. (Peterson, 2021b)

Peterson's position throughout the conversation could not be more emphatic: in the context of our turbulent modernity, psychedelics might be a necessary catalyst in revivifying the religious life of civilization and thereby restoring humankind's rightful orientation toward the cosmos. Far from questioning the inevitability of dominance hierarchies, Peterson easily connects current concepts from the psychedelic research discourse—including the prosocial value of experiencing interconnection—back to his preexisting, hierarchy-based ideology.

According to Peterson's worldview, serving the “good” requires a hierarchical approach to life's challenges, such that the most competent and efficient problem-solvers rise to the top and attain positions of authority within society (Peterson, 2021a). From this perspective, intersectional social justice activism threatens the core mechanism whereby suffering is most efficiently ameliorated, since feminists are attempting to dismantle all hierarchies by labeling them as abuses of power. In conversation with Griffiths, Peterson attempts to correct this supposed misidentification of hierarchy with tyrannical power by emphasizing hierarchy's righteous authority as a form of “reciprocal ethic”:

You hear all these postmodern critiques...of hierarchical structure because of its predication on power; and I think—no, no! *Corrupt* hierarchies are predicated on power. *Functional* hierarchies are predicated...on reciprocity—on *productive* reciprocity…. I was talking to... Jocko Willink, who was the commander of Fallujah... 20 years ago, and he's a real warrior type, you know? He talked about his Navy Seal training, and he said, “Well, we were taught—it was pounded into us to have the back of the guy next to us.” It wasn't like every powerful, clambering ape for himself. Not at all! In these intensely competitive hierarchies, which would be—you'd think—as pure a manifestation of the power motive as would be possible, power is not the guiding ethos. He said quite clearly, “No—Your men won't attend to you unless it's reciprocal….” They have to know *you have their backs*. (Peterson, 2021b)

Peterson suggests that this sense of hierarchy as a “reciprocal ethic” operates cross-culturally with the functional status of “natural law”:

Hierarchies are organized around an ethical principle [as opposed to brute power] if they're to be stable and productive across long spans of time. And…that pattern emerges cross-culturally. It's [based on] reciprocal productivity…. And so, I think you can make a very deep biological case for the…emergent evolution of an ethical sense. And…I think that does speak to people in the voice of their conscience. (Peterson, 2021b)

Peterson theorizes that just as “cocaine hyperstimulates the psychomotor stimulant system,” psychedelics might amplify existing processes in the nervous system—except that, in the case of psychedelics, those processes involve connecting to the dominance hierarchy's reciprocal ethic: “People come out [of the psychedelic experience]—[and] they're more prosocial. And so, [do] you think that's…an exaggeration, or a strengthening, of…our inbuilt, reciprocal ethic?” (Peterson, 2021b).

Unambiguously, Peterson attributes the prosociality induced by psychedelics to grateful acceptance of one's place within the dominance hierarchy—a perspective that he situates within Griffiths' discussion of psychedelics and meaning-making:

[Griffiths:] There's a level of meaning-making that comes out of these experiences that end[s] up rewriting the personal narrative that [a] person has about themselves and about their place in the world, and that account[s] for these enduring changes, and the fact that people then become behaviorally much more flexible; because if that narrative structure is changed…the analogy I think of is [that] you're rewriting the underlying operating system. (Peterson, 2021b)

While Griffiths' perspective on meaning-making is a commonplace interpretation of the evidence-based psychedelic research literature, Peterson reinterprets it in a manner that is compatible with his preexisting conservative political assumptions. Peterson communicates these assumptions by responding with an extended analysis of the Biblical story of Cain and Abel, which he interprets as a parable of the “grievance politics” that he associates with social justice activism. In Peterson's telling, Cain represents those who seek to destroy the dominance hierarchy due to resentment over one's subaltern status. Lacking the wisdom of understanding how hierarchy is in everyone's best interest, these types seek to undermine (or, in Cain's case, to kill) the competent achievers of higher status—even at the expense of increasing their own suffering—out of a misplaced sense of rebelling against injustice:

I think of the operating system as [having] a narrative structure…. The [narrative psychedelic] rewrite seems to me to be something like the revelation...that things are interconnected, that things are fundamentally good…[which] gives some experiential weight that can be used as a counterposition against that destructive cynicism. And the psychedelic, the mystical experience seems to allow for that transformation. (Peterson, 2021b)

Peterson sees the psychedelic insight as shifting personal identification away from injustice toward the interconnected whole of hierarchy, which justifies experiences of oppression and makes them meaningful. Psychedelics reveal “the *meaningful* interconnectedness” of all people and all things, within which “the entire pattern of connection has some transcendent or ultimate significance that's hidden from us” (Peterson, 2021b).^2^ The individual's suffering under hierarchy is sanctified in the name of this transcendent, organic whole—a conceptual framing that Peterson uses to justify the perpetuation of societal inequalities across the categories of class, race, and gender.^3^

While Peterson attempted to distance himself from the alt-right in the aftermath of the Charlottesville rally, this framework—paired with Peterson's emphatic embrace of psychedelics—is likely to speak to the right-wing authoritarian elements of his audience in the coming years. Peterson's discussion with Griffiths about the psychedelic insight that death is a “reparative mechanism” with a “restorative role,” along with his assertion that “if death isn't regulated properly within us, we get cancer,” could be warped by those alt-right elements to serve as justifications for weeding out the “undesirable” elements of society. Later in his discussion with Griffiths, Peterson applies this metaphor to society at large:

But…there's this benevolent death that's a reparative mechanism, and when I've allowed my intuitions to extend themselves as far as possible, I think, well…that's true of…Being itself...is that, in a manner that we can't comprehend, death plays a restorative role…. It's something like the precursor to resurrection, and God only knows where that idea goes. (Peterson, 2021b)

This line of thought connects with a related claim by the adjacent public speaker Jonathan Pageau, a reactionary acolyte of Peterson. Pageau concurs with Peterson's concept of benevolent death as a reparative mechanism and characterizes social justice activists as working to subvert it:

If in traditional societies we see scapegoat mechanisms sacrificing the exception in order to preserve coherence, here it is a desire to sacrifice the entire world for the exception. On a social level, this appears as an upside-down hierarchy, where the strange, the impure, the exceptional, the fluid, the rejected, the sick, and the unknown become a new measure by which society is evaluated. (Pageau, 2021; Thought Slime, 2021)

When “undesirables” become coded as cancer, invasive social measures—including eugenics and genocide—risk becoming justifiable.^4^

## RED Pills for Sale: Neoreaction, Right-Wing Billionaires, and Corporadelic Dreams

Although Peterson invoked fascist metaphors to opine about psychedelics, members of the far right had already co-opted a colorful drug metaphor to talk about the radicalization process: taking the red pill. Under the pen name Mencius Moldbug, Curtis Yarvin was the first to associate anti-democratic, neofascist politics with the metaphor from the sci-fi film *The Matrix* (Moldbug, 2007). Two years later, in the first chapter of Unqualified Reservations (UR), a blog about the neoreactionary (NRx hereafter) movement, Moldbug compared his anti-egalitarian, pro-corporate governance views to red pills—this time in direct comparison to a DMT trip, described in terms that make it clear the author was familiar with the drug's effects (Moldbug, 2009):

There is no such thing as a gentle introduction to UR. It's like talking about a ‘mild DMT trip.’ If it was mild, it wasn't DMT. UR is a strange blog: its goal is to *cure your brain*. We've all seen *The Matrix*. We know about red pills. Many claim to sell them. You can go, for example, to any bookstore, and ask the guy behind the counter for some Noam Chomsky. What you'll get is blue pills soaked in Red #3.

The red pill idiom has since been adopted by incels, neo-Nazis, members of the Trump first family, and billionaires alike (Swearingen, 2020).

Accelerationist philosopher Nick Land's post-academic writings engaged with Moldbug's NRx aspirations, coloring them sufficiently that the NRx project of balkanizing the world into hyper-capitalist city-states is also known as The Dark Enlightenment, after Land's eponymous 2014 essay. During his time at Warwick University, Land co-founded the Cybernetic Culture Research Unit (CCRU), an unofficial project of the philosophy department which orbited around rave culture, post-structuralism, the occult, and technology. Land left academia amid suspicion of providing drugs to students (Whitaker, 2020). Land's vision of acceleration—the idea that capitalist processes should be vastly empowered and hastened in order to bring about dramatic social and political change—evolved to embrace authoritarian, radical right-libertarian ends (Land, 2017), but the philosophy has both right and left (Fisher, 2014) applications. Land's work has been more directly influential on neo-Nazi groups, with altright.org cofounder (with Richard Spencer) Jason Reza Jorjani among them (Goldhill, 2017). Moreover, accelerationism has been appropriated by neo-Nazi circles, inspiring James Mason's *Siege* writings, which have been collected and published by psychedelic-adjacent neo-Nazi musician and journalist Michael Moynihan, whose wife and bandmate, Annabelle Lee, has translated psychedelic works by Albert Hofmann and Christian Rätsch (Piper, 2015). In the 1980's, Mason was in communication with members of the Manson family and today has direct contact with the aforementioned Atomwaffen. Helter-Skelter, Manson's apocalyptic prophecy, was essentially right-wing accelerationism—the murders by the Manson Family were carried out in hope of provoking a race war (Lexington County Chronicle, 2020).^5^

Although Silicon Valley's interest in disruption differs from the overt violence of mass racial attack, the frequent result of market-based disruption is catastrophic upheaval for the marginalized communities. Corporadelic actors are currently folding psychedelics into a corporate ethos predicated on novelty and cognitive labor: from microdosing coders to ayahuasca business coaches, psychedelics are seen as shortcuts to divergent market insight in a globalized, neoliberal marketplace. Following atai Life Sciences' successful IPO, founder and Chairman Christian Angermayer said on CNBC's *Squawk Box*: “The world we're building is a bad place for our brain, so mental health issues will go up. But I do think we have some real shots in our portfolio to end the mental health crisis” (Costa and Shead, 2021). Yet despite publicly discussing his experience with psilocybin and his desire to improve the world, he also directly supports authoritarianism in his advertised resume: Angermayer is on the presidential advisory board of Rwandan head of state Paul Kagame, known internationally for human rights abuses and domestic political repression (Human Rights Watch, 2020; Angermayer, 2021). One of the defining features of authoritarian regimes is unchecked human rights abuses. Since the violence that we do to each other tends to cause the most severe cases of PTSD—more so than natural disasters such as hurricanes or tsunamis—it is unlikely that Angermayer's efforts to spread psilocybin therapy will be an adequate substitute for widespread social change (Kessler et al., 2017). Since billionaires are invested in preserving the status quo of massive wealth inequality, their enthusiasm for psychedelics suggests that this class of drugs is not inherently a solution for ameliorating the extensive harms caused by social inequality.^6^ As described by Tim Ferris, a multi-millionaire psychedelics donor and coach for attaining “expert status...in less than four weeks” (Day, 2018): “The billionaires I know, almost without exception, use hallucinogens on a regular basis” (Fink, 2015). Both Steve Jobs and Bill Gates have admitted to LSD use (Playboy, 1994). Minecraft inventor and billionaire Markus “Notch” Persson is a vocal opponent of feminism, gay pride, and trans acceptance while simultaneously supportive of QAnon and self-experimentation with LSD (Notch, 2017). Tech billionaire Peter Thiel has also invested $12 million in atai Life Sciences and was an early investor in COMPASS Pathways, a company plagued with accusations of seeking to monopolize and patent every conceivable application for psychedelics, including elements of prior art (Hausfeld and Nickles, 2021; Love, 2021; McDaniel, 2021).

Thiel has not definitively acknowledged psychedelic use, but his investment conveys interest, and his politics are decidedly authoritarian: he has stated his ideal business strategy is the formation of a monopoly (Thiel, 2014). Theil's Stanford peer wrote an essay detailing his allegedly unapologetic support for South African apartheid: “He said, with no facial affect, that apartheid was a sound economic system working efficiently, and moral issues were irrelevant” (Lythcott-Haims, 2016). He has also been a vocal and monetary supporter of President Trump and Senator Ted Cruz. Thiel co-founded the surveillance capitalism firm Palantir, which aggregates personal data about private citizens and makes a high-resolution profile easily available to corporate clients (e.g., hedge funds), government agencies like the US Immigration and Customs Enforcement (ICE), police, and militaries. Palantir's billionaire co-founder and CEO Alex Karp has acknowledged that their technology is sometimes used in targeted assassinations (HBO, 2020). Around the same time that Moldbug, described as “[t]he supreme Sith Lord of the neoreactionaries” (Land, 2013), compared his NRx blog to a potent tryptamine, Thiel wrote a disquieting piece for CATO Unbound (a right-wing libertarian think tank funded by billionaire brothers Charles and David Koch). In addition to lamenting women's suffrage and hinting at his own recreational drug use—he opened the piece with: “I no longer believe that freedom and democracy are compatible” (Thiel, 2009). At the end of the essay, Thiel explicitly recommends Moldbug's UR as suggested reading. Moldbug and Thiel share more than corporate monarchist ideology: in 2011 Thiel's Founders Fund provided $100,000 in start-up capital for Moldbug's software company Tlon, where his official title was “benevolent dictator for life” (Pein, 2014; Tait, 2019). Undoubtedly, the kind of corporate feudalism advocated by Thiel and Moldbug is an updated form of digitally-empowered fascism (Smith and Burrows, 2021).

The merger between fascism and big data is nowhere better exemplified than with Cambridge Analytica (CA), the artificial intelligence (AI) company co-founded by billionaire Robert Mercer and former Trump strategist Steve Bannon (Mayer, 2017; Cadwalladr, 2018). Highly targeted voter data scraped from social media sites including Facebook allowed CA to focus on, for example, Catholics who regularly attend mass and voted in the previous election. Such granularity has been credited with swaying results rightward in both Brexit and the 2016 US presidential vote. Both the Mercer Family and Bannon have been the subject of targeted lobbying by psychedelic reformers at the Multidisciplinary Association for Psychedelic Studies (MAPS), enacted directly by former MDMA PTSD therapy recipient and MAPS employee (MAPS, 2018), self-described “conservative whisperer,” Sgt. (R) Jonathan Lubecky (Lubecky, personal communication). MAPS has stated that Lubecky's lobbying work included arranging for placement of a favorable story about MAPS' clinical trials with veterans in the white nationalist mouthpiece, Breitbart News (Bernstein, 2017; Kessler, 2017; Nickles, 2020), which is co-owned by Robert Mercer's daughter, Rebekah Mercer (Church, 2017). Rebekah Mercer is reportedly highly active in the editorial process at Brietbart, to the extent of reading every story and “call[ing] when there are grammatical errors or typos.” Rebekah Mercer has since committed to donating $1 million to research on MDMA therapy for veterans with PTSD. According to social media posts by Lubecky and Rick Doblin's description of Lubecky's work,^7^ his contact with the American political right wing is far-ranging and includes figures such as former President Donald Trump, former Vice President Mike Pence, Rudy Gulliani and Alex Jones (Ferriss, 2020; Nickles, 2020). A year prior to her donation to MAPS, Rebekah Mercer founded Parler (Benveniste and Yurieff, 2020), a social media platform used by many of those who stormed the United States Capitol Building (Cameron and Mehrorta, 2021).

## Addressing the Trojan Horse in the Room

The argument for psychedelic medicalization is made in dollars and cents—MDMA for PTSD is projected to be more cost-effective (Marseille et al., 2020) and profitable than standard treatments. When articulating a broader vision beyond legalizing psychedelic therapy, psychedelic advocates frequently describe a strategy consistent with a Trojan horse theory of change: broader psychedelic use will catalyze geopolitical reconciliation and become the chemical feedstock of world peace. Most recently, MAPS Executive Director Rick Doblin advanced the hypothesis that the mystical experience could be used as “a tool of political reconciliation.” In an interview with spiritual leader and former Democratic Presidential candidate Marianne Williamson (The Marianne Williamson Podcast, 2021), Doblin argued that “humanity is in a race between consciousness and catastrophe.” To wit, the consciousness to which Doblin refers is that of religious fundamentalists. Doblin asserts that the psychedelic experience is the true religion, hoisting a banner under which a global Pax Psychedelia can be negotiated:

I would say that the key challenge I see going forward with the psychedelic renaissance, and also with [the] survival of humanity, is that we have to really help fundamentalists of all the different religions move to a more mystical orientation, and I think that is our challenge. And psychedelics can help that, to see that…what people take literally are metaphors and are symbols; and when you concretize them and…make them literal. And then you are fearful [that] people are infidels, or they don't believe it. So, I think back to America now where we got the Christian fundamentalists…as a major force: how do we help them see that there is a deeper spirituality, that they can have a richer life by surrendering some—And it's not just Christian fundamentalists, it's Orthodox Jews who are crazy in their own way too. And…orthodox Muslims [also].” (The Marianne Williamson Podcast, 2021)

Indeed, Doblin has been making similar claims for most of his career, having once proposed a project entitled “Shaping a Global Spirituality While Living in the Nuclear Age” (Klein, 1985) to the United Nations, which articulated such a vision. Doblin is far from the only psychedelic advocate making sweeping claims about the power of psychedelics to potentiate systemic change, rather than being assimilated and put to use by the current political and economic order (e.g., Peck, 2020).

But if Doblin's gambit is that ingestion of psychedelics by evangelical Christians will help them find “a deeper spirituality,” the case of close Trump ally and “active member” of the First Baptist Church Matt Gaetz (R-FL) is a stubborn datapoint (Kaylor and Underwood, 2021). Although Gaetz co-sponsored an (unsuccessful) amendment to remove a bill rider that inhibited therapeutic research into Schedule I controlled substances such as psilocybin, MDMA and marijuana (Jaeger, 2019), it is now plain that the Representative's interest in psychedelics was not solely legislative. Evidence has since surfaced that he allegedly used MDMA himself during encounters with sex workers (Bort, 2021). Famously, Gaetz supported drug-testing welfare recipients (Vlamis, 2021), demonstrating that conservatives may support psychedelics for medical or recreational purposes, but they will likely continue to defend wealth-based hierarchy.

## Psychedelic Cultures of Authoritarianism and Violence

Even if the policy goals of psychedelic reformers come to fruition, they will still have to reckon with the authoritarianism and violence that have haunted psychedelic-assisted therapy since its inception. In Torsten Passie's history of MDMA, he cites Deborah Harlow's account of how the male-female therapeutic dyad arose as a safeguard against therapists sexually manipulating their drugged clients (2018). Despite these historical roots, subsequent scientific accounts of the dyad have emphasized its psychoanalytic rationale (e.g., providing a mother and father figure to facilitate developmental transference) while omitting warnings about the danger of therapist abuse: “With a male-female dual-therapist model, the therapists hold different transferential roles during the session and are often secure attachment figures available to the patient for contact and bonding” (Penn et al., 2018).

This rebranding of the therapeutic dyad does little to institute meaningful safeguards for the exaggerated power imbalance inherent in psychedelic therapy. The case of the psychedelic researcher who facilitated a MAPS MDMA PTSD Phase II therapeutic trial, Richard Yensen, is illustrative. Yensen was chaperoned by his wife and dyad counterpart Dr. Donna Dryer in recorded sessions with patient Meaghan Buisson. Nevertheless, Yensen used his authority to initiate a multi-year sexual relationship with Buisson that was counter-therapeutic and predatory (Goldhill, 2020; Lindsay, 2021).

A recent Mad in America article, “Breaking the Silence Around Psychedelic Therapy Abuse,” details many examples of authoritarian mistreatment and assault by influential practitioners—some of whom remain influential and promoted in the field (Hall, 2021). Citing Salvador Roquet, an early psychedelic therapist, Hall points to a lineage of practice wherein authoritarian methods are encouraged through formal, underground training. Assault has been a problem in other environments where vulnerable, psychedelicized people are supervised by unscrupulous authority figures, as in the therapy-adjacent shamanic and neoshamanic contexts described by Ross (2018). Ross' subsequent research has demonstrated how dominant approaches to sexual abuse in psychedelic contexts rely on neoliberal narratives about individual responsibility. These narratives avoid the systemic nature of power imbalances magnified by vulnerability and suggestibility (Ross, 2020).

Within hierarchical organizations, these dynamics have been weaponized by powerful, charismatic figures for the purposes of influencing group behavior. Among new religious movements, a still-living founder's perceived omniscience, esoteric knowledge, or prophetic ability can promote an extremist worldview of “absolute righteousness” where “killing becomes healing” (Strozier, 2010). For example, psychedelics played a role in both the Aum Shinrikyo and Rajneesh cults, which committed terrorist acts against unsuspecting civilians using chemical and biological weapons (LeHardy, 1997). Members of both groups were intimately involved with psychedelic manufacture and use. Aum Shinrikyo began as a small yoga school in the mid-1980s, attracting New Age spiritual seekers with teachings directed toward “enlightenment” and “awakening” (Maekawa, 2001). Many top leaders in Aum Shinrikyo admitted to using LSD in ritual practices “to stimulate spiritual activity,” and psychedelics were also administered to Aum's recruits as part of the conditioning and indoctrination process (Kogo, 2002; Thornton, 2017). In 1995, members of Aum Shinrikyo intentionally released the chemical nerve agent sarin in the Tokyo subway, killing 14 people and injuring 5,500. The same Aum scientist who produced its sarin gas was also responsible for producing “research quantities” of LSD for the cult (Danzig et al., 2012; Thornton, 2017).

Cult violence in the New Age was prefigured by the Rajneesh movement in the decade prior. The sanyassins, as its followers were called, frequently had experience with LSD before joining the group as there was significant overlap between psychedelic culture and the movement's spiritual ideals (Palmer and Sharma, 1993). Many sanyassins were apprehended internationally on drug trafficking charges. According to its own members, Rajneeshee activities were at least partly funded by drug smuggling, and drug trafficking was a major source of income for the movement (McCormick, 1987). At least one follower was directly involved with LSD manufacture: prolific LSD chemist Nick Sand lived and worked in several sanyassin ashrams, first moving to Pune, India to be closer to Bhagwan Rajneesh. Sand bought a house near the ashram, where he built a lab and manufactured “massive” quantities of LSD. Sand also claimed that he helped Rajneesh get a passport to move to Oregon (Scully, 2017). It was in Oregon where the inner authoritarianism of the group bled out into the wider world, when sanyassins attempted to influence a local election by driving down voter turnout with a bioterror attack on a salad bar.

## Conclusions

Experiments or studies that have sought to explore whether psychedelics have a consistent impact on political ideology and psychedelic use have thus far failed to rule out lurking variables in participant set and setting. Researchers should be careful to avoid conflating single-issue beliefs with specific ideologies, since particular values (e.g., environmentalism) are represented across different political perspectives and shift over time. We discourage the use of underpowered studies to make broad claims, especially given the risk of exaggeration and distortion in the media, which can further influence patient expectation and researcher bias (Carhart-Harris et al., 2018). Because of the sensitivity of study participants to suggestion under the influence of psychedelics, it is crucial that researchers publish the manuals used in psychedelic therapy and describe any significant variation within the study of the therapeutic environment (e.g., the presence or absence of religious imagery) (Lattin, 2021). Since public relations campaigns, intake procedures, questionnaires, and preparatory sessions can all prime and influence participant expectations, which can in turn affect a study's measured outcomes, we recommend the development of new standards for reporting on psychedelic clinical trials that include open publication of these materials (Petranker et al., 2020; Muthukumaraswamy et al., 2021).

Assertions that psychedelics induce a shift in political beliefs will have to address the many historical and contemporary cases of psychedelic users who remained authoritarian in their views after taking a wide variety of psychedelics or became radicalized after extensive experience with them. We propose that anecdotal accounts of psychedelics precipitating radical shifts in political or religious beliefs are common but result from factors relating to the set and setting and have no inherent directional basis on the axes of conservatism-liberalism or authoritarianism-egalitarianism. Instead, we draw from critical perspectives in the humanities and social sciences to argue that any experience which challenges a person's fundamental worldview—including psychedelic experience—can precipitate shifts in political belief.

Whether on grounds of supporting the troops or following doctors' orders, psychedelics—properly contextualized—are palatable to conservatives. In fact, themes of psychedelic authoritarianism within the context of medicalization go beyond the PR campaigns of organizations such as MAPS. Such authoritarian tendencies have appeared in clinical trials, underground contexts, and instances of state-sanctioned psychedelic torture (Nickles, in press). Additionally, several modern conservative cultural icons are on record as supportive of psychedelic medicalization. During a conversation on The Rubin Report—an IDW-affiliated interview show—Jordan Peterson, conservative ideologue Ben Shapiro, and host Dave Rubin discussed the spiritual and medical benefits of psilocybin and MDMA. Peterson admits to experience with psilocybin (The Duncan Trussell Family Hour, 2017) and Rubin confirms experience with both. While pressing the uninitiated Shapiro about the merit of these drugs, Shapiro concedes that he would be supportive of their use, but only if under the care of a physician:

I think the answer is that it depends on, you know, the level of expertise of the person using it is what I would suggest, you know, the idea—like, I'm not against prescription medication, so if you're talking about, you know, an actual program that's going to better somebody's life that's one thing, but if it's somebody who's just ‘I want to have a religious experience, let me pop some LSD’ then—there's some serious downsides to drugs. (The Rubin Report, 2018)

To Shapiro, the authority of a medical provider would be enough to overcome his personal reticence about currently illegal psychedelic substances. For Peterson and Rubin, there is no evidence that their psychedelic experiences present a barrier to their activities—not only as conservatives, but as elite thought leaders of the conservative cultural project.

As institutional support for psychedelic therapy grows, hierarchy-conscious conservatives are likely to shift their opinions about the acceptability of taking psychedelics themselves. Within the field of psychedelic studies, the non-specific nature of the interface between psychedelics and ideology is a positive opportunity for interdisciplinary scholarship on the cultural plasticity and suggestibility inherent to psychedelic experiences:

As the project of mainstreaming the uses of psychedelic drugs is proving surprisingly successful, these substances also enter into forms of life that are very different from the ones that have fostered the revival [of institutional psychedelic research]. While liberal and left-leaning anthropologists might find this pluralism...disturbing..., it's a boon to the anthropology of psychedelics. Studied ethnographically, rightist psychedelia might teach us a great deal about the cultural plasticity of the relationship between human beings and these wondrous molecules. (Langlitz, 2020)

The fact that members of reactionary and right-wing authoritarian political movements make their own uses of psychedelics is an opportunity to broaden theoretical claims about psychedelic mechanisms, which can help researchers to avoid making uncritical and simplistic generalizations based on the limited data of existing studies. This is critical to acknowledge as simplistic narratives about the social benefits of psychedelics are being trumpeted by profit-motivated actors. As corporate speculation on psychedelic medicine booms, commentators have seized on the potential for psychedelics to disrupt a mental health industry valued at $100 billion (Yakowicz, 2021). Although much of this discourse focuses on individualistic therapeutic effects, a recurring theme is the notion that psychedelics can solve complex social problems by making people more politically liberal (Adams, 2020).

While the notion of individual solutions for systemic problems has been widely critiqued across academic disciplines, we believe that the common narratives regarding the effects of psychedelics on political orientation present persistent blind spots within the psychedelic literature. It is imperative to address these blind spots to advance coherent, interdisciplinary socio-political frameworks for analyzing and engaging with the experiential realities and potential implications of psychedelic drug use.

## Data Availability Statement

The original contributions presented in the study are included in the article/supplementary material, further inquiries can be directed to the corresponding author.

## Author Contributions

BP and ND contributed to the research conception, drafting of the article, critical revision of the draft, and final approval of the manuscript. Both authors contributed to the article and approved the submitted version.

## Funding

Funding from the Ohio State University's Department of Plant Pathology is committed to paying publication fees.

## Conflict of Interest

The authors declare that they are unpaid board members of Psymposia, a 501c3 nonprofit research organization.

## Publisher's Note

All claims expressed in this article are solely those of the authors and do not necessarily represent those of their affiliated organizations, or those of the publisher, the editors and the reviewers. Any product that may be evaluated in this article, or claim that may be made by its manufacturer, is not guaranteed or endorsed by the publisher.

## Footnotes

^1^Group norms famously vary: large-scale ritual human sacrifice of the Nahua Aztecs and Mayans occurred in a context of regular consumption of both mescaline and psilocybin (Carod-Artal, 2015; Jay, 2019). Some members of the Northwestern Amazonian Jivaro-Shuar have used ayahuasca for war-making where the brew is used specifically to prepare the user to fight and improve their killing abilities (Rubenstein, 2012; Gearin and Devenot, 2021).

^2^Peterson's embrace of interconnection with hierarchy has deep roots in the “Western” intellectual tradition, which suggests that it was only a matter of time before the popularization of psychedelics united these ideological positions. His ideas are reminiscent of those expressed by Alexander Pope during the English Enlightenment in “An Essay on Man” (1733), which used ideas about the Great Chain of Being (an idea that was newly reinvigorated by the development of the microscope and telescope, which revealed animate worlds beyond the ordinary bounds of human vision) to justify the status quo of hierarchical power relations across society:
See, through this air, this ocean, and this earth, All matter quick, and bursting into birth. Above, how high, progressive life may go! Around, how wide! how deep extend below! Vast chain of being…. ...what no eye can see, No glass [telescope] can reach! from infinite to thee, From thee to nothing!—On superior pow'rs Were we to press, inferior might on ours: [...] Where, one step broken, the great scale's destroy'd: From nature's chain whatever link you strike, Tenth or ten thousandth, breaks the chain alike. [...] The least confusion but in one... ...the whole must fall. Let earth unbalanc'd from her orbit fly, Planets and suns run lawless through the sky; Let ruling angels from their spheres be hurl'd, Being on being wreck'd, and world on world; Heav'n's whole foundations to their centre nod, And nature tremble to the throne of God. All this dread order break—for whom? for thee? Vile worm!—Oh madness, pride, impiety! [...] Cease then, nor order imperfection name: Our proper bliss depends on what we blame. Know thy own point: This kind, this due degree Of blindness, weakness, Heav'n bestows on thee. Submit.— [...] All nature is but art, unknown to thee; All chance, direction, which thou canst not see; All discord, harmony, not understood; All partial evil, universal good: [...] One truth is clear, Whatever is, is right. (Pope, 1891) Pope implies in “An Essay on Man” that “slavery is not a situation imposed from outside but a moral condition emanating from within” (Richardson, 2003), and that—as with Peterson's Cain—refusal to gratefully accept one's place within an interconnected hierarchy is to threaten the very structure of the cosmos itself.

^3^From Peterson's perspective, psychedelics thus help to teach a lesson that he earlier described in *12 Rules*: when you aspire to greatness within the hierarchy, “you disturb others, in the depths of their souls, where they understand that their cynicism and immobility are unjustifiable. You play Abel to their Cain. You remind them that...they do not want to lift the world up on to their shoulders, where it belongs” (Peterson, 2018).

^4^Although Jordan Peterson is not advocating for genocide, the logic of coding social undesirables in public health terms informed the “racial hygenics” that justified Nazi policy: “A credo repeatedly articulated by the National Socialists held that what was wrong in Germany could be powerfully and usefully expressed through medical analogy. The ‘body’ of the German people was threatened, and its healer was Hitler. As anti-Semitism was added to social Darwinism and racial hygiene, the so-called Jewish question was rendered a medical problem, ‘the therapy of which was to be realized in places like Auschwitz and Dachau’.... Gradually, the medical profession embraced the belief that to cure individual persons was one thing, but to heal the nation was incomparably more important; thus, most in the profession adopted the ideas of racial hygiene as a massive public health measure. ‘The aim of generating pure Aryans had taken precedence over the most fundamental ethical issues in medicine’...and healing acquired a new, sociopolitical definition that swept aside the vulnerability and suffering of large numbers of individual persons, who, in a sense, lost their humanity and were transmuted into pollutants of the state” (Barondess, 1998).

^5^Matthew Johnson cites Manson's manipulative influence over his psychedelicized subordinates as an extreme example of the dangers of imposing one's beliefs on others in a psychedelic setting (Johnson, 2021).

^6^Commentators and psychedelics researchers have a tendency to ignore the structural realities and incentives of capitalism while arguing that psychedelic experiences of interconnection will inspire billionaires to act for the betterment of society. This perspective parallels arguments for sending billionaires to space, in order to facilitate an “overview effect” that inspires social change. Amazon Executive Chairman Jeff Bezos suggested that his trip into low Earth orbit increased his concern for Earth's “fragile” ecosystem; however, the solutions he has offered thus far are still predicated on the market logic of infinite growth with extractive industries. Rather than questioning whether feelings of interconnection are sufficient for systemic social change, Johns Hopkins psychedelics researcher David Yaden speculated that Bezos might not have achieved the overview effect with just 10 minutes in low orbit (Woodward, 2021).

^7^As Doblin stated on The Tim Ferriss Show: “And after our treatment, he was able to go back to work and he was actually the liaison to the veterans for Senator Rand Paul for his 2016 presidential nomination campaign for the Republican nomination. And so when I met Jon, he was doing much better, but he also had political connections. And so I felt that that's really what we need to broaden the support for what we're doing to have a veteran who's got connections with a lot of Republicans who is willing to speak about the benefits of MDMA. And so actually Jon has met with Vice President Pence, with Stephen Miller, with all sorts of Republicans that he was able to speak with and have, in a way, the first impressions that they get about MDMA and the potential of MDMA to help prevent suicides among veterans, to help veterans come back and lead more healthy lives. They would hear about it from Jon. So now he's working as a consultant for MAPS to try to broaden our political support.”
